# Chats about Medical Museums

**Published:** 1887-04-09

**Authors:** 


### Chats about Medical Museums.

By One of the Crowd.

III.—Royal College of Surgeons

(*Concluded from page* 391).

An extensive museum such as that of the Royal College of Surgeons, to which reference was made in the first article under this heading, is admirably adapted to impress the average visitor with a sense of his own ignorance. At every turn and on all hands there are objects which cannot but suggest to the mind questions, to which the greatest profundity of scientific lore would be able to supply no answer, and which, to those of us whose profundity is not very great, are apt to prove somewhat humiliating. It is not perhaps altogether agreeable to have one's ignorance thus displayed to one's own gaze ; but no doubt it is wholesome. At any rate, most persons of sense and sensibility who stroll round a place like this, must find it inevitable, and there are probably few who do so who do not long for a Huxley or an Owen to act as cicerone and expositor.

In the absence of such a guide, philosopher, and friend, the uninstructed stranger will probably do best to wander here and there, as his inclination leads him, and pick up such crumbs of interest and enlightenment as may fall in his way. If he be a bit of a phrenologist —as we most of us are, consciously or unconsciously— he will be greatly interested in a series of casts representing the interior of various skulls, intended to furnish an idea of the size and form of the brain in various races of man, and in different groups of the mammalia. The simpler plan, it may be thought, would be to present casts of the brain itself ; but this is usually impracticable, from the fact that the brain is so soft in its texture, that it does not preserve its shape after removal from the body. The interior of the cavity of the skull, however, corresponds very closely with the form of the brain, and a cast of this cavity, with certain slight allowances for the thickness of membranes, may be taken accurately to present the brain in its size and form. But perhaps the point of most striking interest in this connection is that which has undoubtedly presented the greatest stumbling-block to the progress of phrenology. The inside shape of these cranial cavities does not correspond with the outside of the skull, as presented to the phrenologist. The "walls" vary in thickness, and there are muscles and air-cells, which give to external "bumps" a prominence and a form which may seriously misrepresent the form of the actual brain within. Still, it is undeniable that in the normal head the general size and shape of the skull may be taken to fairly indicate the size and shape of the brain. There are in this collection some preserved heads of natives of the New Hebrides, who appear, like some of the North American Indians, to have had their foreheads flattened back in infancy. It may, perhaps, be regarded as another difficulty in the way of phrenology, that this curious practice of artificially malforming the skull and the brain within it, does not appear to have exerted any influence on the mental faculties of tribes who have practised it. So, at all events, it has been said by some who have had opportunities of observing.

On the ground-floor of the eastern museum there is much to interest the naturalist, in the shape of skeletons of various kinds of animals. Those who are not naturalists, may find at least one very curious object in a portion of the bow of Her Majesty's ship *Fawn*, which has been pierced by a species of sword fish. Such a terrible thrust has the assailant given with the prolonged upper jaw that has given him his name, that he has driven his unlucky snout through the copper sheathing, the felt, the deal, and the hard oak timber of the ship to the depth of fourteen inches. The creature had probably never met with a British man-of-war before, and had no idea what it was made of. He acquired some little information for subsequent use, but he broke his proboscis, and left more than a foot of it in the ship's timbers, and doubtless retired a sadder if a wiser swordfish. The fibres of the oak timber, as the catalogue points out, are bent and crushed as by the passage of a swivel-ball. In this chamber will be found the skeleton of the first gorilla brought to this country. This acquisition made a considerable sensation in scientific circles some five-and-thirty years ago. Artemus Ward would have called him a mean-looking cuss as he stands here in his glass case, so much like a human being as inevitably to invite a comparison between himself and the skeleton of his more respectable relatives in the other room. He has short lower limbs, and this makes him decidedly the inferior of an average man in point of stature, but he has a far larger trunk than an ordinary man, and a pair of arms of such length and development as to indicate enormous muscular power. He is a much nicer animal in the *déshabillé* of bare bones in a glass case than he could ever have been to look in the face in his native Gaboon woods.

Passing up the staircase at the inner end of the western museum one comes at the top to a gruesome little room containing some plaster casts exhibiting various abnormal conditions of the human body, perhaps the most remarkable of which is that of a man with a horn growing from the middle of his forehead. This protuberance bends down the whole length of the middle of his face, and their is another promising little sprout in the middle of his cheek. Another cast—presumably taken from the head of a living man of mature age—represents two faces grown together. There are only two eyes, but there is a double supply of nose, and two mouths and two chins. One face is larger than the other, but, as might be expected under the circumstances, there is a strong family likeness. In going up to this little chamber, one passes, on the landing below, a water-colour back and front drawing of the body of a man, who survived an accident which, if he had not been the most unreasonable and irrepressible of mortals ever born, ought to have killed him on the spot. He was one John Taylor, a Prussian, on board a ship in the London Docks in 1831, when some tackle he was manipulating gave way, and the pivot of the try-sail mast went clean through his body—in at his chest and out at his back, passing obliquely across between the heart and the left lung. It could not have been a very big thing, the reader will think—that pivot of the try-sail mast. But here lies the very thing itself under the drawing representing the man's wounds. It is a massive spike of iron, perhaps sixteen inches long and an inch and a half thick. After this it looks perfectly childish for any man to succumb to a mere bullet. Not only was this invincible individual thus perforated, but his scalp was laid open, and his lower jaw and four of his ribs were fractured. The fellow would not die, however. He went to the London Hospital for five or six months, and eventually returned to his duty as a seaman, and more than once afterwards visited the College of Surgeons in a robust state of health. Another case of a very similar kind is represented by the shaft of a chaise, standing in the corner by the side of the object just mentioned, and by a "preparation," in another part of the museum, showing the chest of a man through whose body the shaft had been driven by a restive horse. In some respects this was a more extraordinary occurrence than the other, and has been deemed worthy of a special treatise, illustrated by diagrams, etc. The shaft is a somewhat pointed one, but yet a horribly blunt and bulky affair for a man to be impaled upon. It is said to have gone clean through his body up to the first tug-hook, which was also driven into his chest so far as to wound the left lung. Nevertheless this man is said to have fully recovered his health and strength, and to have lived eleven years after the injury. This took place in 1812, since when how many men have died from the scratch of a pin or a puff or two of cold air?

(*To be continued.*)

